# Pregnancy with Concomitant Chorioangioma and Placental Mesenchymal Dysplasia: A Rare Placental Abnormality

**DOI:** 10.1155/2013/591956

**Published:** 2013-06-10

**Authors:** Wu Qichang, Wang Wenbo, Zheng Liangkai, Kong Hui, He Xiaoqin, Sun Li, Xu Yasong

**Affiliations:** Prenatal Diagnosis Center of Xiamen's Maternal & Child Health Care Hospital, Xiamen, Fujian 361000, China

## Abstract

*Background*. Pregnancy with concomitant chorioangioma and placental mesenchymal dysplasia (PMD) coexisting with a normal viable fetus is very rare. The literature was reviewed to explore the incidence and genetic origin of this condition. *Case*. The case was first identified by prenatal ultrasonography, but the prenatal diagnosis only included chorioangioma. PMD was then confirmed during postnatal evaluation, which included gross and histologic examination of the placenta. The macroscopic and microscopic findings were consistent with concomitant chorioangioma and placental mesenchymal dysplasia during pregnancy. Genetic findings confirmed genetic similarity of the chorioangioma and vesicle-like villi with the fetus. *Conclusions*. The case represents a rare placental abnormality whose pathogenesis and molecular basis need further research. Detailed histologic and genetic analyses are essential for accurate and differential diagnosis.

## 1. Introduction

Chorioangioma, a benign vascular tumor arising from chorionic tissue, is the most common tumor of the placenta [1]. Large lesions negatively affect outcome for both the mother and fetus [2]. Placental mesenchymal dysplasia (PMD), an abnormality with a diverse etiology, is becoming more widely recognized [3]. Pregnancy with concomitant chorioangioma and placental mesenchymal dysplasia represents a rare combination of abnormalities. In this report, we present a case in which concomitant chorioangioma and placental mesenchymal dysplasia coexisted with and were genetically identical to a normal viable fetus. We also reviewed the literature exploring the underlying cause of PMD.

## 2. Case Report

**Figure 1:**
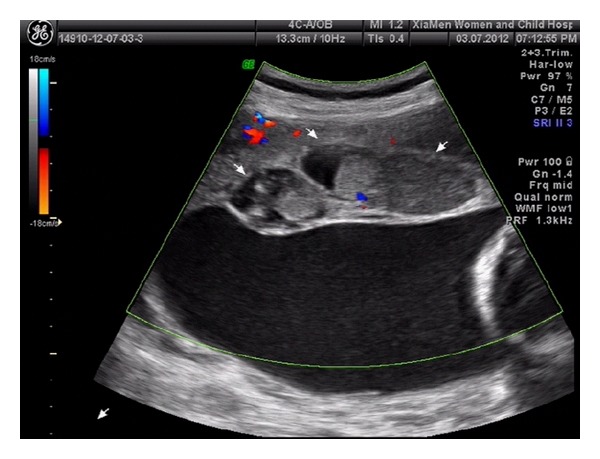
Transabdominal ultrasound showing a bulky placenta contained a mixed-echogenic mass measuring approximately 83 × 30 × 56 mm.

**Figure 2:**
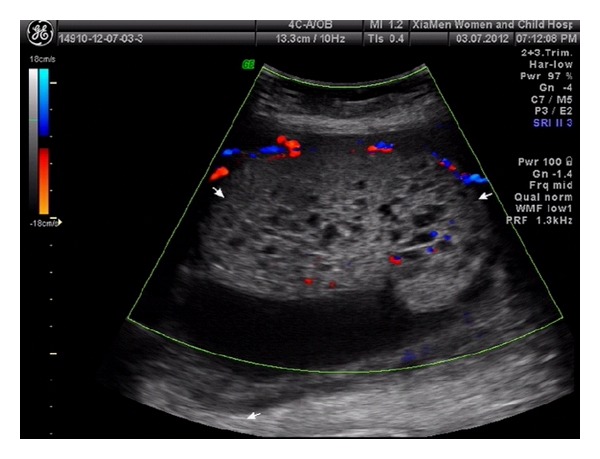
Placenta with multiple cystic echoes (such as moth-eaten appearance) without vascular flow.

**Figure 3:**
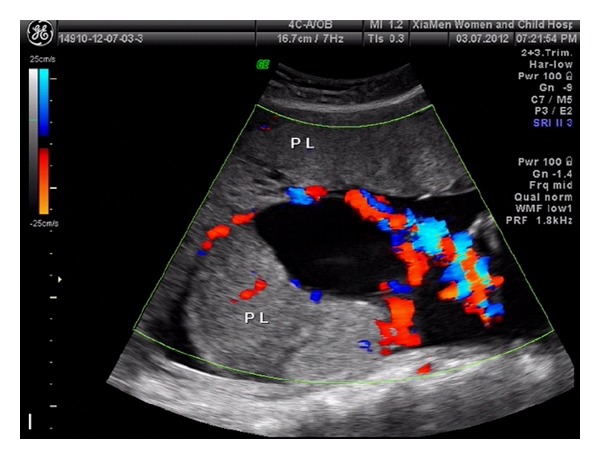
Ultrasonography shows a circumscribed echogenic mass measuring 115 × 110 × 112 mm containing anechoic cystic areas with pulsatile blood flow. The mass protrudes from the fetal surface of the placenta into the amniotic cavity.

An asymptomatic 30-year-old Chinese primigravida underwent her initial routine ultrasonography examination at 25 weeks of gestation. Sonographic examination confirmed a structurally normal fetus with an estimated gestational age of 25 weeks. The placenta appeared bulky and contained a well-defined 83 × 30 × 56 mm mixed-echogenic mass (Figure 1) and areas of multiple cystic echoes without vascular flow giving the mass a moth-eaten appearance (Figure 2); in particular, a well-circumscribed echogenic mass, measuring 115 × 110 × 112 mm and containing anechoic cystic areas, protruded into the amniotic cavity from the fetal surface near the cord insertion (Figure 3). On color Doppler images, the anechoic areas indicated pulsatile blood flow within the intrauterine masses. The largest amniotic fluid pocket measured 7.5 cm, and amniotic fluid index equaled 23.5 cm. The sonographic appearance of the masses and the presence of polyhydramnios suggested a prenatal diagnosis of placental chorioangioma, but the significance of the multiple cystic echoes in the placenta was neglected. The presumptive diagnosis and potential outcome were discussed with the patient. Because the tumors were large and multiple, we suggested referral to a specialized maternal-fetal medicine department for interventional management that could include serial fetal transfusions and fetoscopic laser coagulation of vessels supplying the tumor. The patient refused this alternative and returned to our hospital at 27^+3^ weeks of gestation in preterm labor. Ultrasonography examination revealed polyhydramnios, with an increase in the amniotic fluid index to 37.5 cm. She was admitted for tocolysis, but this management was unsuccessful. One day after admission a 740 g female was delivered vaginally with Apgar scores of 5 and 8 at 1 min and 5 min, respectively. The maternal serum *β*-human chorionic gonadotropin (*β*-hCG) was 4611 milli-international units/mL at 2 days postpartum and was undetectable at 3 weeks postpartum.

**Figure 4:**
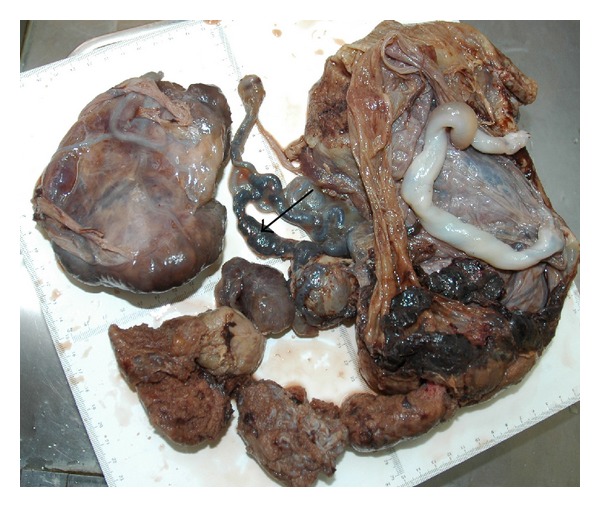
Photograph of the gross specimen of the placenta; the placenta measured 30 × 25 × 4.5 cm and weighed 1370 g, and the largest tumor measured 11 × 8 × 4.5 cm. Arrow pointing to the fetal plate showed enlarged varicose chorionic vessels.

**Figure 5:**
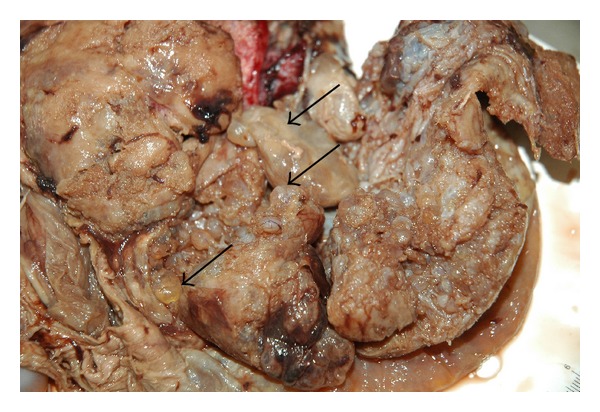
Macroscopic features of the placenta show that the maternal plate was filled with diffuse vesicle-like structures. (arrows).

**Figure 6:**
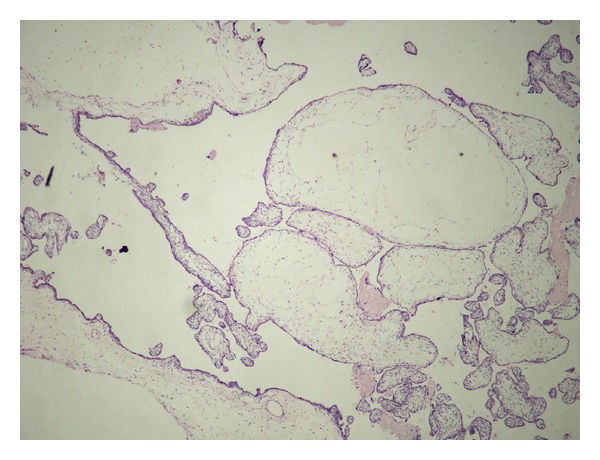
Photomicrograph showing large, edematous stem villi without trophoblastic proliferation. Hematoxylin and eosin stain. Original magnification ×10.

**Figure 7:**
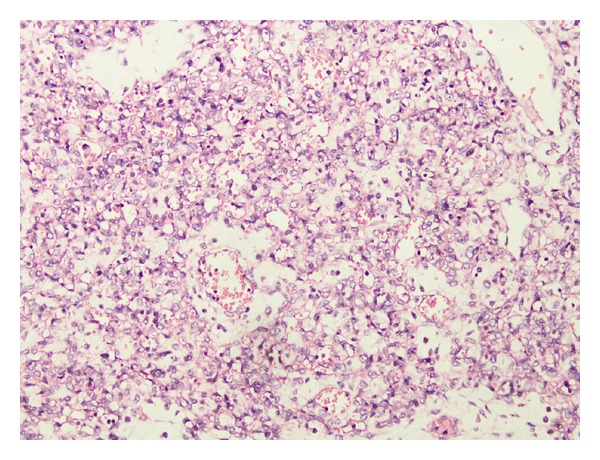
Microscopic examination of chorioangioma with lakes of blood vessels suggestive of angiomatous pattern. Hematoxylin and eosin stain. Original magnification ×40.

**Figure 8:**
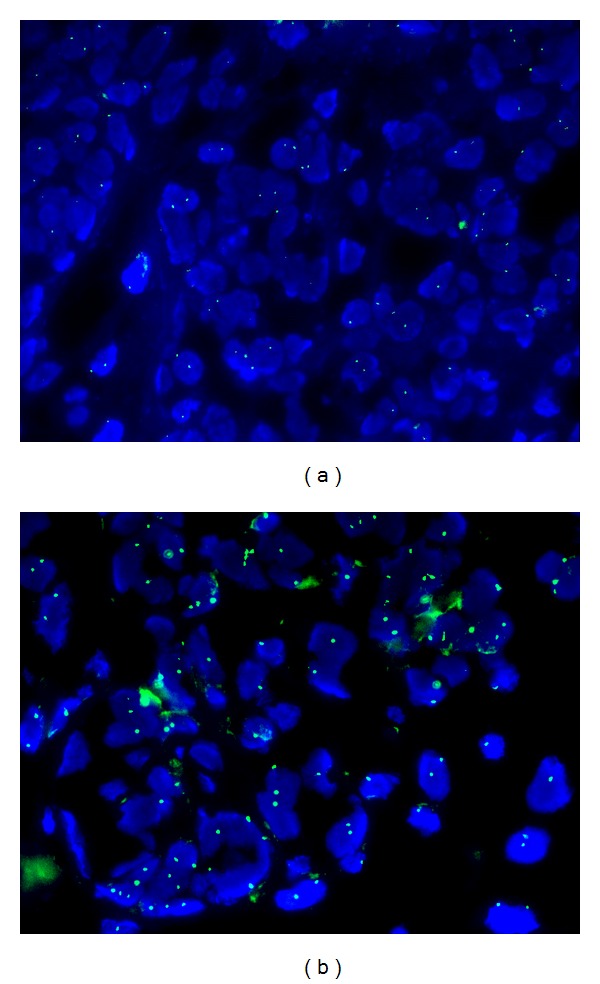
Interphase fluorescent in situ hybridization (FISH) image showing DAPI stained in the vesicle-like stem villi and the chorioangioma. The nuclei were hybridized with a green X-centromere (DXZ1) and a red Y-centromere probe (DYZ3). Only green X-centromere (DXZ1) probe were found within a single cell.

**Figure 9:**
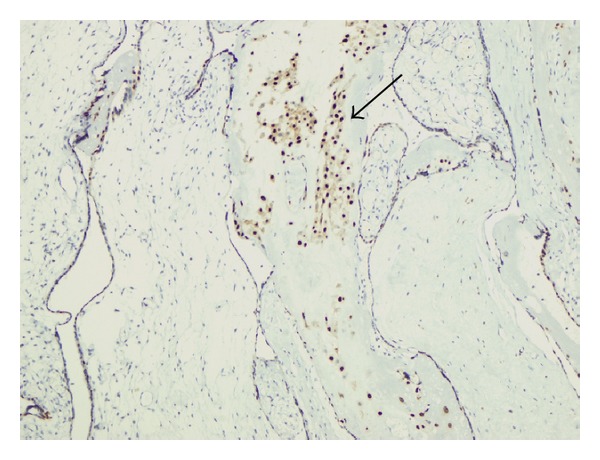
p57^KIP2^ positivity in extravillous trophoblast nuclei (original magnification ×10).

The placenta measured 30 × 25 × 4.5 cm and weighed 1370 g, and the largest tumor measured 11 × 8 × 4.5 cm (Figure 4). The fetal plate showed enlarged varicose chorionic vessels (Figure 4). The maternal plate was filled with diffuse vesicle-like structures (Figure 5). The clinical macroscopic features were similar to partial molar pregnancy, but on histopathologic examination, no trophoblastic proliferation was seen in the placenta (Figure 6). The diagnosis of chorioangioma was confirmed histologically in the masses. The masses were classified as angiomatous (capillary) chorioangioma, which is the most common histologic type (Figure 7). The fetal karyotype was 46,XX. Molecular genetic analysis of the vesicle-like stem villi and the chorioangioma using FISH techniques revealed the same 46,XX karyotype (Figure 8). Expression of p57^KIP2^ in the villous cytotrophoblast was investigated by immunohistochemical examination with Streptavidin-Peroxidase (SP) (Figure 9). On the basis of these findings, a final diagnosis of pregnancy with concomitant chorioangioma and placental mesenchymal dysplasia was made.

## 3. Discussion

Pregnancy and a live birth with concomitant chorioangioma and PMD are very rare. To our knowledge, only 3 cases of concomitant chorioangioma and PMD have been reported [4]. The rarity of this condition and the interest in its genetic origin and pregnancy outcome prompted this report and our review of the literature. 

Placental mesenchymal dysplasia (PMD) was first described in 1991 by Moscoso et al. [5] and has since then become a more widely recognized vascular anomaly. The hallmarks of PMD are placentomegaly and grape-like vesicles resembling partial molar pregnancy on ultrasonography and gross placental examination [6]. The underlying cause of PMD is currently unknown. Some speculate that PMD is a congenital malformation of the mesoderm. This theory is based on observations of mesenchymal hyperplasia in stem villi along with other placental mesenchymal proliferative disorders including chorioangiomas and chorionic vessel dilatation as well as hemangiomas of the fetus [7]. The theory suggests that the combination of PMD and chorioangioma represents a mixed form of congenital malformation of the mesoderm. 

No specific clinical symptomatology is associated with PMD. However, PMD has been associated with fetal growth restriction, stillbirth, and Beckwith-Wiedemann syndrome [7]. Large or multiple chorioangiomas have a particularly dismal prognosis due to their high association with maternal and fetal complications, including polyhydramnios, preterm labor, fetal hemolytic anemia, fetal thrombocytopenia, cardiomegaly, intrauterine growth restriction, placental abruption, preeclampsia, and congenital abnormalities [2]. Our case was complicated by polyhydramnios, preterm labor, and prematurity, consistent with the above-mentioned findings. 

PMD should be considered in the differential diagnosis of cystic placenta. Ultrasonographic features of this abnormality closely resemble those of partial hydatidiform mole. The coexistence of a large cystic placenta with a phenotypically well-formed fetus is highly unlikely in molar pregnancy and should increase the possibility of a PMD diagnosis. The 2 entities are distinguished primarily by karyotype analysis. Partial moles are triploid, whereas PMD carries a 46,XX karyotype [3]. In the present case, the neonate as well as the vesicle-like stem villi and the chorioangioma shared the 46,XX karyotype. These findings confirmed those of Chen et al., who reported similar genetic identity among the chorioangioma, vesicle-like villi, and fetus [7]. Partial moles also display trophoblastic proliferation, a feature not seen in PMD. In the present case, no trophoblastic proliferation was noted on microscopic examination of the tumors, thereby establishing the diagnosis of PMD.

A review of the literature indicates that approximately 25% cases of PMD are associated with Beckwith-Wiedemann syndrome (BWS), a condition characterized by macrosomia, exomphalos, macroglossia, omphalocele, internal visceromegaly, placentomegaly, and increased susceptibility to childhood tumors [8]. The pathogeneses of BWS and PMD may be similar, with the strongest evidence pointing toward epigenetic imprinting abnormalities involving genes on chromosome 11p15.5. The genetic alterations found in BWS and implicated in PMD may be part of a spectrum, with gene aberrations found solely in the fetus (BWS), localized to the placenta (PMD), or both (PMD plus BWS) [9]. The most commonly involved genes are CDKNIC (p57^KIP2^), H19, IGF-II, and KVLQT [6]. There have been no major analyses of the frequency of these molecular alterations in PMD. Immunohistochemical staining for the p57^KIP2^ protein as a diagnostic marker may prove helpful in distinguishing PMD from molar pregnancy [10]; however, this cannot be confirmed if the underlying 11p15.5 abnormality is confined to the placenta. Further research to detect PMD-specific gene abnormalities associated with BWS, using microarray or microsatellite technology or both, may be helpful.

The molecular basis of the placental changes in PMD remains unclear. The presence of 2 distinct cell lines in the placenta of some patients with PMD suggests androgenetic-biparental mosaicism as an etiology [3]. The androgenetic cell line is believed to arise from endoreduplication of the haploid paternal genome, whereas the biparental cell line arises from the combination of the haploid maternal and the paternal genomes. This could also explain the female predominance of the condition. 

Many pathologists are unfamiliar with PMD, which may account for the underdiagnosis and underreporting of this entity. Our case documents the salient features of PMD and the distinguishing features from those disorders mimicking this condition, the most important of which is a partial mole.

In conclusion, pregnancy with concomitant chorioangioma and placental mesenchymal dysplasia is very rare. Our case illustrates the importance of a careful gross and histologic examination of the placenta in making an accurate diagnosis. Our findings confirm those of others and suggest that chorioangioma and PMD have the same pathologic origin. Both are derived from the mesoderm, and in our case, the chorioangioma, vesicle-like villi, and the fetus shared an identical genetic profile. However, the pathogenesis and the molecular basis of PMD need further research.
